# Defense Responses of Different Rice Varieties Affect Growth Performance and Food Utilization of *Cnaphalocrocis medinalis* Larvae

**DOI:** 10.1186/s12284-024-00683-2

**Published:** 2024-01-20

**Authors:** Xiaoyu Zhao, Hongxing Xu, Yajun Yang, Tianyi Sun, Farman Ullah, Pingyang Zhu, Yanhui Lu, Jianlei Huang, Zhengliang Wang, Zhongxian Lu, Jiawen Guo

**Affiliations:** 1 State Key Laboratory for Managing Biotic and Chemical Threats to the Quality and Safety of Agro- Products, Institute of Plant Protection and Microbiology, Zhejiang Academy of Agricultural Sciences, Hangzhou, 310021 China; 2 College of Life Sciences, China Jiliang University, Hangzhou, 310018 China; 3 College of Plant Protection, Nanjing Agricultural University, Nanjing, 210095 China; 4 College of Life Sciences, Zhejiang Normal University, Jinhua, 321004 China; 5 College of Agriculture and Forestry, Hebei North University, Zhangjiakou, 075000 China

**Keywords:** Rice Varieties, *Cnaphalocrocis medinalis*, Growth and Development, Nutritional Effect Index, Defensive Response

## Abstract

Rice leaf folder, *Cnaphalocrocis medinalis* (Guenée), is one of the most serious pests on rice. At present, chemical control is the main method for controlling this pest. However, the indiscriminate use of chemical insecticides has non-target effects and may cause environmental pollution. Besides, leaf curling behavior by *C. medinalis* may indirectly reduce the efficacy of chemical spray. Therefore, it is crucial to cultivate efficient rice varieties resistant to this pest. Previous studies have found that three different rice varieties, Zhongzao39 (ZZ39), Xiushui134 (XS134), and Yongyou1540 (YY1540), had varying degrees of infestation by *C. medinalis*. However, it is currently unclear whether the reason for this difference is related to the difference in defense ability of the three rice varieties against the infestation of *C. medinalis*. To explore this issue, the current study investigated the effects of three rice varieties on the growth performance and food utilization capability of the 4th instar *C. medinalis*. Further, it elucidated the differences in defense responses among different rice varieties based on the differences in leaf physiological and biochemical indicators and their impact on population occurrence. The results showed that the larval survival rate was the lowest, and the development period was significantly prolonged after feeding on YY1540. This was not related to the differences in leaf wax, pigments, and nutritional components among the three rice varieties nor to the feeding preferences of the larvae. The rate of superoxide anion production, hydrogen peroxide content, and the activity of three protective enzymes were negatively correlated with larval survival rate, and they all showed the highest in YY1540 leaves. Compared to other tested varieties, although the larvae feeding on YY1540 had higher conversion efficiency of ingested food and lower relative consumption rate, their relative growth was faster, indicating stronger food utilization capability. However, they had a lower accumulation of protein. This suggests that different rice varieties had different levels of oxidative stress after infestation by *C. medinalis*. The defense response of YY1540 was more intense, which was not conducive to the development of the larvae population. These results will provide new insights into the interaction mechanism between different rice varieties and *C. medinalis* and provide a theoretical basis for cultivating rice varieties resistant to this pest.

**Supplementary Information:**

The online version contains supplementary material available at 10.1186/s12284-024-00683-2.

## Introduction

Plants typically initiate unique defense responses after being attacked by herbivorous insects (Fürstenberg-Hägg et al. 2013). The rapid production of reactive oxygen species (ROS) is one of the early defense responses of plants to external stimuli (Kerchev et al. 2012; Mittler et al. 2022). Superoxide (O_2_^−^) and hydrogen peroxide (H_2_O_2_) are the most important ROS in response to insect infection (Thorpe et al. 2013). However, the accumulation of excessive ROS can lead to cell damage or death by oxidizing lipids and forming lipid peroxides such as malondialdehyde (MDA) (Abbasi et al. 2007; Hussain et al. 2018; Nouman et al. 2014). The steady state of ROS in cells is achieved through a balance between production and clearance (Miller et al. 2008). To prevent oxidative bursts caused by ROS production, plants have evolved complex protective mechanisms to eliminate ROS. Superoxide dismutase (SOD), peroxidase (POD), and catalase (CAT) are enzymatic antioxidant systems that regulate the homeostasis of ROS in organisms (Cheah et al. 2015). These enzymes participate in O_2_^−^ reduction to H_2_O to eliminate excessive ROS, and this ROS clearance mechanism plays an important role in plant–insect interactions (Cheah et al. 2015; Gill and Tuteja 2010; Nouman et al. 2016). Several studies have reported that ROS play a key role in defending plants against insects. For example, ROS are involved in the defense of wheat and rice against *Mayetiola destructor* larvae and in the egg deposition response of *Pinus sylvestris* against *Diprion pini* (Bittner et al. 2017; Liu et al. 2010). However, investigating reactive oxygen species (ROS) in the context of plant defense against insects is still preliminary.

Rice (*Oryza sativa* L.) is one of the most important food crops in the world. Various pests infest this important crop during its growth period (Liu et al. 2016). In response to the invasion of pests, rice develops complex defense reactions to affect the adaptability of pests and reduce the degree of damage (Kumar et al. 2022; Nanda et al. 2020). Previous studies have found that the degree of infestation by rice pests such as *Oebalus pugnax* F. and *Nilaparvata Lugens* is closely related to rice varieties (Bhavanam and Stout 2022; Cheng et al. 2013; Ojha and Zhang 2019). Different rice varieties have different defense responses to pest infestation (Hu et al. 2018; Shangguan et al. 2018). For example, the SOD activity of Pf9279-4 was significantly higher than that of 02428 after infestation by *N. Lugens* (Dong et al. 2017). The activities of SOD and POD in insect-resistant rice (TE358, TE363, TE367) increased, while CAT activity decreased following infestation by *Sogatella furcifera* (Zhang and Xue 2004). The defense response of different rice varieties usually has various effects on indicators such as pest growth, food utilization, and reproduction (Alamgir et al. 2016; Antunes et al. 2016; Peñalver-Cruz and Horgan 2022).

The rice leaf folder, *Cnaphalocrocis medinalis* (Guenée), is one of the most serious pests on rice (Han et al. 2021). *C. medinalis* larvae feed on rice leaves, especially during the grain-filling stage, which affects the photosynthetic capability of rice and seriously threatens the safety of rice production (Guo et al. 2019b). At present, chemical control is the main method for controlling *C. medinalis* larvae (Sun et al. 2023). However, the indiscriminate use of chemical insecticides has non-target effects and may cause environmental pollution (Desneux et al. 2007; Tang et al. 2021). In addition, *C. medinalis* larvae build a feeding chamber by folding a leaf longitudinally with silk, indirectly protecting them from the chemical spray (Cheah et al. 2020). Therefore, it is crucial to screen and cultivate efficient rice varieties resistant to *C. medinalis*. Several studies reported that the occurrence of *C. medinalis* varies among different rice varieties (Alinia et al. 2000; Liu et al. 2012; Xu et al. 2010). These defense differences will affect the survival, growth, and reproduction of *C. medinalis* (Amb and Ahluwalia 2016; Yadava et al. 1972). Correspondingly, *C. medinalis* generates various defense signals based on its various defense responses, promoting further adaptation and development of the population (Cheah et al. 2020; Guo et al. 2019b). Our previous field experiments have found that the occurrence of *C. medinalis* is different in three different rice varieties, including Zhongzao39 (ZZ39), Xiushui 134 (XS134), and Yongyou 1540 (YY1540) (Fig. S1). However, it is unclear whether these occurrence variations are directly linked to the differences in defense mechanisms among the three rice varieties against *C. medinalis* larvae. Based on previous studies, we speculate that different rice varieties had different levels of defense response after infestation by *C. medinalis*, which may lead to differences in the accumulation of ROS and related enzyme activities, physiological and biochemical indicators in their leaves, thereby affecting growth performance and food utilization ability of larvae.

Therefore, the current study was conducted to clarify the differences in defense responses of three rice varieties and their effects on the growth performance and food utilization capability of *C. medinalis* larvae. This study investigated the effects of three different rice varieties on the survival rate of different instars, larval developmental duration, food utilization capability, and nutrient accumulation of the 4th instar. We observed the feeding preferences of larvae towards three rice varieties. Moreover, we measured the differences in physiological and biochemical indicators such as wax, pigment, nutrient content, antioxidant enzymes (SOD, POD, CAT), MDA, O_2_^−^ production rate, and H_2_O_2_ content in the leaves of different rice varieties. The correlation between these indicators was also analyzed to clarify the differences in defense responses among different rice varieties and their effects on the occurrence of *C. medinalis* population. These results will provide new insights into the interaction mechanism between different rice varieties and *C. medinalis* and provide a theoretical basis for breeding rice varieties resistant to this key pest.

## Materials and Methods

### Rice Plant Preparing and Insect Rearing

The seeds of three rice varieties, YY1540 (Ningbo Seed Co., Ltd, Ningbo, China), XS134, and ZZ39 (Zhejiang Wuwangnong Seeds Shareholding Co., Ltd, Hangzhou, China), were soaked for 24 h and drained, and then covered with moist gauze to induce germination for 48 h (Zhao et al. 2022). They were planted in a white plastic bowl (26 cm × 17 cm × 8 cm in length, width, and height) to raise seedlings and transferred to flower pots (diameter 12 cm) after 12 d. All three varieties were planted in the greenhouse at Zhejiang Academy of Agricultural Sciences in Hangzhou, China (30.31° N, 120.20° E), and were used for experiments 40 d after transplantation. There was no pest feeding or pesticide treatment during rice growth.

*C. medinalis* larvae were collected from Nanjing, Jiangsu Province, in 2019 (118.78° E, 32.06° N) and were reared on wheat seedlings until pupation (Guo et al. 2022). Pupae were transferred into a plastic box (16 cm × 24 cm × 22 cm), and the bottom of the box was covered with moist cotton for moisture retention. After emergence, six females and six males were transferred to a 500 mL plastic cup with 5% honey solution-soaked cotton at the bottom, which was sealed with cling film (used to collect eggs).

All the insects were reared in RXZ intelligent artificial climate chambers (Ningbo Jiangnan Instrument Factory, Ningbo, Zhejiang, China) at 26 ± 1 °C, 60 ± 5% relative humidity, and a photoperiod of 14:10 L:D (Guo et al. 2022).

### Survival Rate and Development Duration of Larvae after Feeding on Different Rice Varieties

Rice leaves (8 cm) at the tillering stage were cut off, and their two ends were wrapped with wet cotton and placed in a culture dish (with a diameter of 12 cm) covered with moist filter paper at the bottom (4 leaves per dish). Five newly hatched larvae were selected and placed in a culture dish. Fresh rice leaves were replaced every day until the larvae pupated. The larval development duration and the number of surviving larvae at each instar were recorded, and the survival rate was calculated. Twenty biological replicates were set for each treatment.

### Feeding Preferences of Larvae on Different Rice Varieties

The feeding preferences of the 1st to 5th instar *C. medinalis* larvae (feeding on wheat) towards different rice varieties were observed. The 1st and 2nd instar larvae were starved for 1 h, and the 3rd to 5th instar larvae for 4 h. The larvae were placed in a 700 mL plastic cup (with a damp filter paper placed at the bottom to maintain humidity). The three sides of the plastic cup were respectively opened to connect three channels (diameter 2 cm, length 11 cm). The rice seedlings are placed at the end of each channel. After 8 h, the total number of larvae on rice was recorded. The number of 1st instar larvae per treatment was 30, 2nd instar larvae was 20, and 3rd to 5th instar larvae was 15. Ten biological replicates were set for each treatment group. The feeding preference was then calculated as:

1 $$ \begin{array}{l}{\rm{Feeding}}\,{\rm{preference}}\, = \,({\rm{the}}\,{\rm{number}}\,{\rm{of}}\,{\rm{larvae}}\,{\rm{on}}\,{\rm{each}}\,{\rm{rice}}\,{\rm{variety}}/\\\,\,\,\,\,\,\,\,\,\,\,\,\,\,\,\,\,\,\,\,\,\,\,{\rm{total}}\,{\rm{number}}\,{\rm{of}}\,{\rm{larvae}}) \times \,100\end{array}$$

### Physiological Characteristics of Different Variety Rice Leaves

#### Wax Content in Leaves of Different Rice Varieties

The leaves of different rice varieties were cut into pieces. 4 g of rice leaves were weighed and placed in a beaker. 60 mL of CHCl_3_ was added and soaked for 1 min. The extract was filtered into a weighed beaker and placed in a fume hood until all CHCl_3_ had evaporated. The beaker was then weighed, and the resulting value was subtracted from the beaker weight to obtain the wax content of the leaves. The experiment was conducted with three biological replicates.

### Pigment Concentration in Leaves of Different Rice Varieties

Various rice leaves were rinsed with distilled water and dried on absorbent paper. After removing the main veins, the leaves were cut into small pieces. Then 0.1 g of rice leaves were weighed and put in a 50 mL centrifuge tube containing 9 mL of mixture (anhydrous ethanol: acetone: water = 4.5:4.5:1). The centrifuge tubes were placed in a dark place overnight and centrifuged at 12,000 rpm for 5 min (Micro 17R, Thermo Fisher Scientific Inc., Karlsruhe, Germany). The absorbance of the supernatant at 663 nm, 645 nm, and 470 nm was measured (SpectraMax 190, Molecular Devices, LLC., San Jose, CA, USA). The mixture was used as a blank control. The contents of chlorophyll a, chlorophyll b, carotenoids, and total chlorophyll were calculated based on the absorbance (Cui et al. 2023). This experiment consisted of 5 biological replicates.

### Effects of Infestation by Larvae on the Nutrient Content of Leaves of Different Rice Varieties

The leaves of three rice varieties were divided into two treatment groups: (i) the group infested by *C. medinalis* larvae: The newly hatched larvae were fed separately with three rice varieties until reaching the 4th instar. Fifteen larvae were taken and fed on new rice plants. The larvae were picked out, and the feces were cleaned with a brush after 48 h. (ii) The uninfested group (CK): rice that larvae had not infested was placed under the same environmental conditions as the above treatment group for 48 h. 0.02 g of rice leaves infested by larvae were weighed for nutrient content determination. Five biological replicates were set for each rice variety treatment group. All samples were frozen in liquid nitrogen and stored at -80 °C for subsequent separation and determination of glycogen, lipid, and protein. The separation of glycogen, lipid, and protein follows Guo’s method (Guo et al. 2019a, 2022). The glycogen, lipid, and protein contents were measured using a microplate reader (SpectraMax 190, Molecular Devices, LLC., San Jose, CA, USA). The specific method was as follows:

The glycogen content was determined using the anthrone spectrophotometric methods: 5 mL of 1 mg/mL anthrone reagent was added to each sample. After mixing, the tubes were heated with boiling water for 10 min and cooled on ice. The absorbance at 620 nm was measured, and the glycogen content was calculated with reference to the glucose standard sample.

The lipid content was determined by the sulfo-phosphoric acid-vanillin method: 1 mL of hexane and 1 mL of concentrated sulfuric acid were added to the sample and then heated in boiling water for 10 min. Cooling the tubes at room temperature, 5 mL of vanillin phosphate solution was added to each tube. The absorbance of samples at 530 nm was measured, and the lipid content was calculated based on the cholesterol standard sample.

Using Pierce ® The BCA Protein Quantitative Analysis Kit (Thermo Fisher Scientific, USA) measured protein content. 150 μL of samples were added 1200 μL WR solution mixed for 30 s and incubated at 37 °C for 30 min. The absorbance of the sample at 562 nm was measured, and the protein content was calculated based on the bovine serum albumin standard sample.

### Effects of Different Rice Varieties on Food Utilization Capability of 4th Instar *C. medinalis*

To ensure the accuracy of food utilization capacity assessment, the food in the larval gut was emptied by starvation before the experiment. Ten 4th instar larvae feeding on different rice varieties were starved for 4 h, and their fresh weight was recorded. Then, the larvae were transferred to a centrifuge tube and dried in a 55 °C oven for 6 h before weighing the dry weight of the larvae. 2 g of rice leaves (tillering stage) from different varieties were wrapped in newspapers and dried in a 55 °C oven for 4 h before weighing the dry weight. In addition, 2 g of rice leaves (with stems) from different varieties were weighed, and the stems were inserted into rubber stoppers. The stem exposed at the bottom of the rubber stopper was wrapped in soaked cotton. The stem was inserted into a 50 mL centrifuge tube containing water. The rubber stopper and centrifuge tube were fixed with a sealing film. Subsequently, they were placed in a 700 mL plastic cup to feed larvae. Ten 4th instar larvae were placed on rice leaves, and the cups were sealed with plastic wrap. After 24 h, the larvae are transferred to a culture dish covered with dry filter paper. The larvae were placed in culture dishes for 4 h to empty the feces in the gut. Larval feces in cups and culture dishes, larvae, and rice leaves cleared of feces were collected and dried to constant weight in an oven at 55 °C. The dry weight of larval feces, larvae, and leaves was recorded. At the same time, an additional 0.2 g of rice leaves were taken and placed in a plastic cup (without larvae). Then, after using the same method and processing time as mentioned above, weigh the dry weight of the leaves to correct the deviation in feed intake caused by water evaporation for the larvae. Each treatment group was set with 5 replicates. The food utilization capability (relative consumption rate (RCR), relative growth rate (RGR), efficiency of conversion of ingested food (ECI), efficiency of conversion of digested food (ECD), and approximate digestibility (AD) of larvae feeding on different rice varieties was calculated according to the following formulae (Jin et al. 2020).

2 $$ \begin{array}{l}{\rm{RCR}}({\rm{g}}/{\rm{g}}/{\rm{d}}) = \\\frac{{{\rm{dry}}\,{\rm{weight}}\,{\rm{of}}\,{\rm{food}}\,{\rm{eaten}}}}{{{\rm{duration}}\,{\rm{of}}\,{\rm{feeding}} \times {\rm{mean}}\,{\rm{dry}}\,{\rm{weight}}\,{\rm{of}}\,{\rm{the}}\,{\rm{larvae}}\,{\rm{during}}\,{\rm{the}}\,{\rm{feeding}}\,{\rm{period}}}}\end{array}$$

3 $$ \begin{array}{l}{\rm{RGR}}({\rm{g}}/{\rm{g}}/{\rm{d}}) = \\\frac{{{\rm{dry}}\,{\rm{weight}}\,{\rm{gain}}\,{\rm{of}}\,{\rm{the}}\,{\rm{larvae}}\,{\rm{during}}\,{\rm{period}}}}{{{\rm{duration}}\,{\rm{of}}\,{\rm{feeding}} \times {\rm{mean}}\,{\rm{dry}}\,{\rm{weight}}\,{\rm{of}}\,{\rm{the}}\,{\rm{larvae}}\,{\rm{during}}\,{\rm{the}}\,{\rm{feeding}}\,{\rm{period}}}}\end{array}$$

4 $$ {\rm{ECI}}\left( \%\right) = \frac{{{\rm{dry}}\,{\rm{weight}}\,{\rm{gain}}\,{\rm{of}}\,{\rm{larva}}}}{{{\rm{dry}}\,{\rm{weight}}\,{\rm{of}}\,{\rm{food}}\,{\rm{eaten}}}} \times 100 $$

5 $$ \begin{array}{l}{\rm{ECD}}\left( {\rm{\% }} \right){\rm{ = }}\\\frac{{{\rm{dry}}\,{\rm{weight}}\,{\rm{gain}}\,{\rm{of}}\,{\rm{larva}}}}{{{\rm{dry}}\,{\rm{weight}}\,{\rm{of}}\,{\rm{food}}\,{\rm{eaten - dry}}\,{\rm{weight}}\,{\rm{of}}\,{\rm{faeces}}\,{\rm{produced}}}}{\rm{ \times 100}}\end{array} $$

6 $$ \begin{array}{l}{\rm{AD}}\left( {\rm{\% }} \right){\rm{ = }}\\\frac{{{\rm{dry}}\,{\rm{weight}}\,{\rm{of}}\,{\rm{food}}\,{\rm{eaten - dry}}\,{\rm{weight}}\,{\rm{of}}\,{\rm{faeces}}\,{\rm{produced}}}}{{{\rm{dry}}\,{\rm{weight}}\,{\rm{of}}\,{\rm{food}}\,{\rm{eaten}}}}{\rm{ \times 100}}\end{array} $$

### Effects of Nutrient Accumulation in the 4th Instar C. medinalis Feeding on Different Rice Varieties

The 4th instar *C. medinalis* feeding on different rice varieties was collected. Each treatment was set with 5 biological replicates, each with five larvae. Samples were immediately frozen in liquid nitrogen and stored at − 80 °C until use and were collected and stored using this method where not specifically described below. The subsequent separation and determination of glycogen, lipid, and protein were the same as in the above methods.

### Effects of Larvae Infestation on O_2_^−^ Production Rate, Content of H_2_O_2_, MDA and Antioxidant Enzyme Activity in Leaves of Different Rice Varieties

The O_2_^−^ production rate, content of H_2_O_2_, MDA, and activities of SOD, POD, and CAT activities were measured to analyze the biochemical responses of various rice leaves to 4th instar larvae of *C. medinalis.* The O_2_^−^ production rate and content of H_2_O_2_ were measured using Superoxide Anion Content Assay Kit and Hydrogen Peroxide (H_2_O_2_) Content Assay Kit (Beijing Boxbio Science & Technology Co., Ltd., Beijing, China), respectively. The determination of MDA content and activities of SOD, POD, and CAT were carried out using Plant Malondialdehyde (MDA) assay kit (Colorimetric method), Superoxide Dismutase (SOD) assay kit (WST-1 method), Peroxidase assay kit and Catalase (CAT) assay kit (Visible light) (Nanjing Jiancheng Bioengineering Institute, Nanjing, China), respectively. Using the above method, 0.05 g of rice leaves from different infested and uninfested varieties by the 4th instar *C. medinalis* were collected. Rice leaves, and 200 μL extract were ground in an ice bath as enzyme sources. The homogenate was centrifuged at 3,500 rpm for 10 min at 4 °C to separate the supernatant. Three biological replicates were set for each group. According to the kit instructions, the absorbance was determined using a microplate reader.

### Statistical Analysis

Before analysis, data were tested for normal distribution and variance homogeneity using the Shapiro–Wilk and Levene tests, respectively. All data conform to normal distribution (*p* > 0.05) and homogeneity of variance (*p* > 0.05). Chi-square test was used to analyze the feeding preference and survival rate (*p* < 0.05). The percentage of food utilization was arcsine square-root transformed before the analysis. The wax and pigment content in leaves of different rice varieties, the nutrient content, O_2_^−^ production rate, H_2_O_2_ content, MDA content and antioxidant enzyme activity in rice leaves, the larval development duration, the food utilization capability, and nutrient accumulation of larvae feeding on different rice varieties were analyzed using Tukey’s honestly significant difference (Tukey’s HSD) tests or *t* test. The correlation between the survival rate, food utilization capability, nutrient content, larval developmental duration, and physiological and biochemical indicators in the leaves of different rice varieties was analyzed using Spearman correlation analysis. All data were statistically analyzed using IBM SPSS Statistics software 26.0.

## Results

### Survival Rate and Development Duration of C. medinalis Larvae after Feeding on Different Rice Varieties

**Fig. 1:**
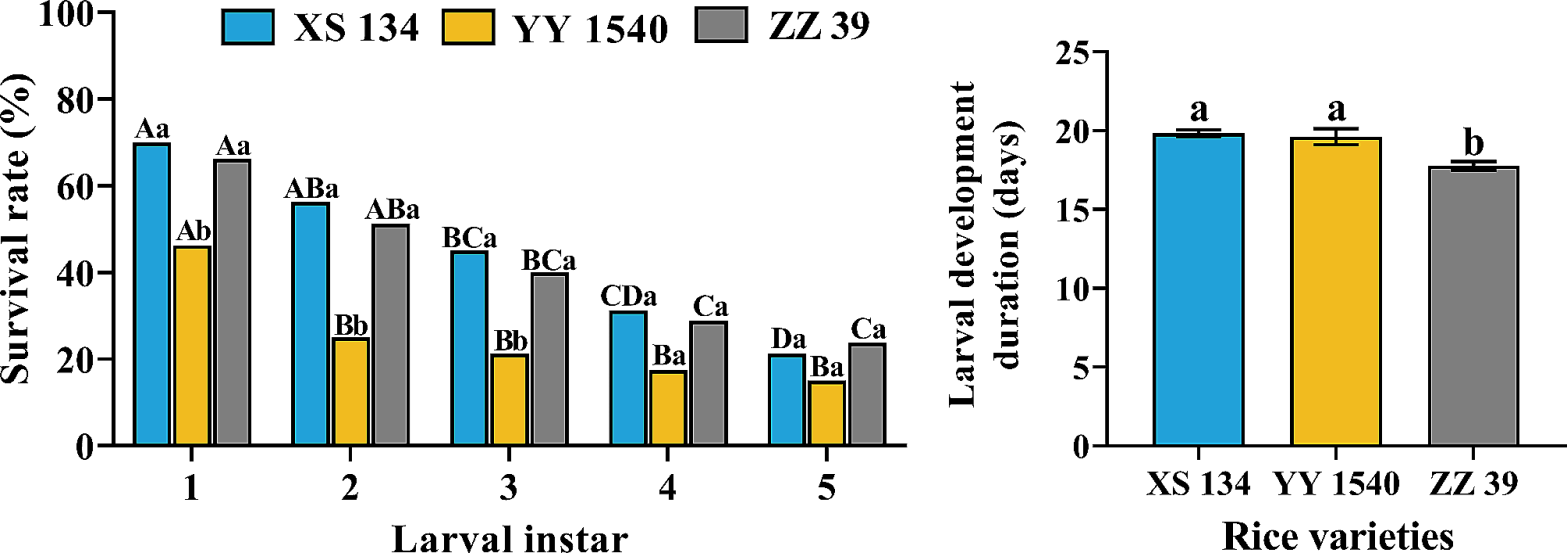
Effects of survival rate at different instars and development duration of *C. medinalis* larvae feeding on different rice varieties. Different lowercase letters indicate significant differences in survival rate (Chi-square test- *p* < 0.05) and larval development duration (Tukey’s HSD- *p* < 0.05) among different rice varieties, and different uppercase letters indicate significant differences in survival rate among different larval instar (Chi-square test- *p* < 0.05)

Regardless of the variety of rice leaves that *C. medinalis* larvae fed on, the survival rate of larvae significantly decreased with the increasing instars (XS134: *χ*^*2*^ = 61.404, df = 4, *p* < 0.001; YY1540: *χ*^*2*^ = 32.320, df = 4, *p* < 0.001; ZZ39: *χ*^*2*^ = 47.373, df = 4, *p* < 0.001) (Fig. 1). The survival rate of the 1st to 3rd instar larvae feeding on YY1540 was significantly lower than other two rice varieties (1st instar: *χ*^*2*^ = 13.857, df = 2, *p* = 0.001; 2nd instar: *χ*^*2*^ = 22.484, df = 2, *p* < 0.001; 3rd instar: *χ*^*2*^ = 14.034, df = 2, *p* = 0.001) (Fig. 1). Although the survival rate of 4th to 5th instar larvae feeding on YY1540 was also lower than other two varieties, this difference was not statistically significant (4th instar: *χ*^*2*^ = 5.094, df = 2, *p* = 0.078; 5th instar: *χ*^*2*^ = 2.625, df = 2, *p* = 0.269) (Fig. 1). However, the developmental duration of larvae feeding on YY1540 and XX134 was significantly longer than that of larvae feeding on ZZ39 (*F*_*2,40*_ = 15.473, *p* < 0.001) (Fig. 1). The larval developmental duration was significantly lower following feeding on ZZ39 compared to other two treatments (Fig. 1). These results indicated that feeding on YY1540 had a more significant inhibitory effect on the survival and development of *C. medinalis* larvae.

### Feeding Preferences of C. medinalis Larvae to Different Rice Varieties

**Fig. 2:**
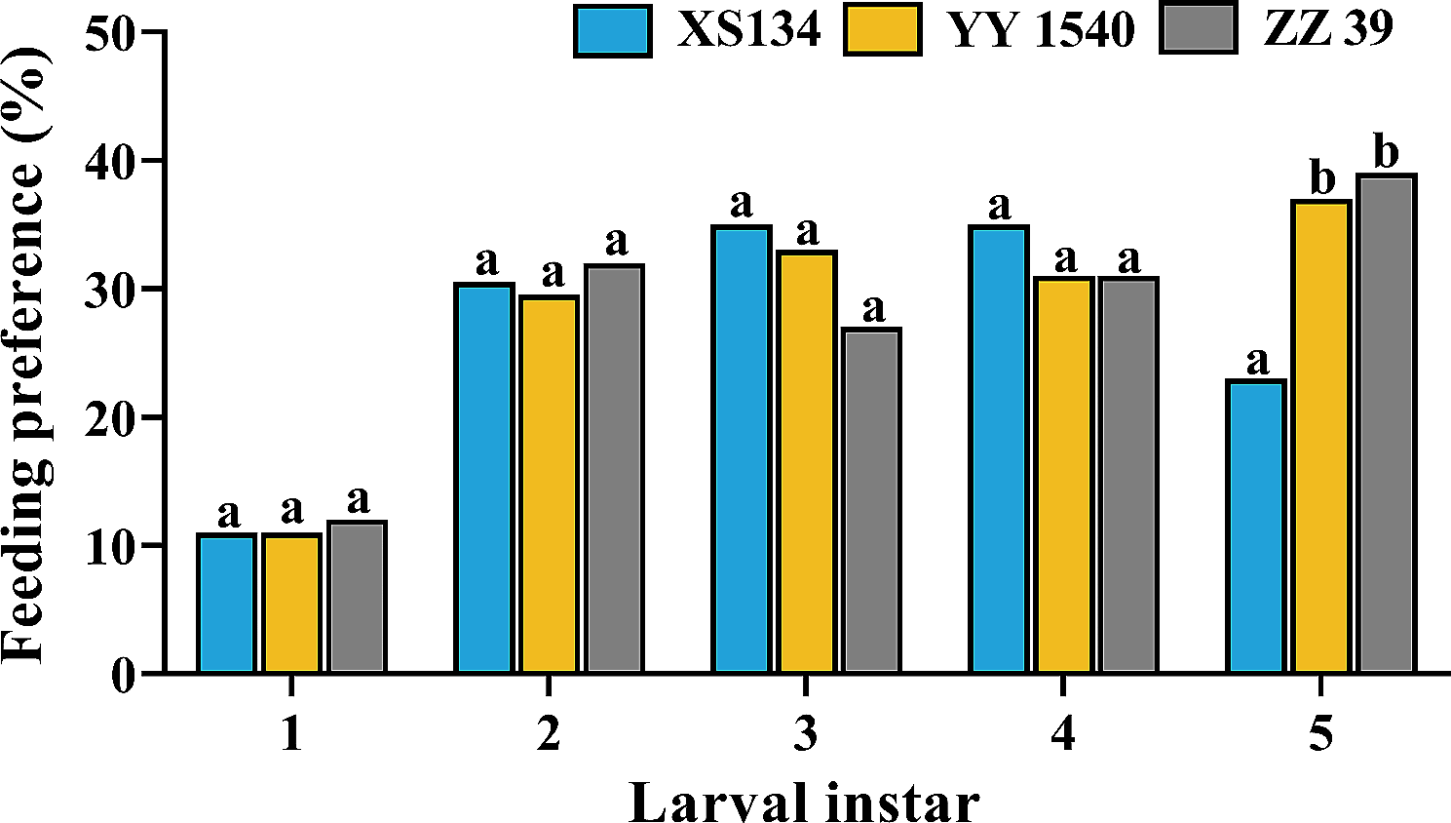
Effects of different rice varieties on the feeding preference of *C. medinalis* larvae at different instars. Different lowercase letters indicate significant differences in the feeding preference among different rice varieties (Chi-square test- *p* < 0.05)

The 1st to 4th instar *C. medinalis* showed no significant feeding preference for the three rice varieties (1st instar: *χ*^*2*^ = 0.204, df = 2, *p* = 0.903; 2nd instar: *χ*^*2*^ = 0.298, df = 2, *p* = 0.862; 3rd instar: *χ*^*2*^ = 1.996, df = 2, *p* = 0.362; 4th instar: *χ*^*2*^ = 0.997, df = 2, *p* = 0.607) (Fig. 2). However, the feeding preference of the 5th instar larvae for ZZ39 and YY1540 was significantly higher than that of XS134 (*χ*^*2*^ = 11.218, df = 2, *p* = 0. 004) (Fig. 2).

### Physiological Characteristics in Leaves of Different Rice Varieties

**Fig. 3:**
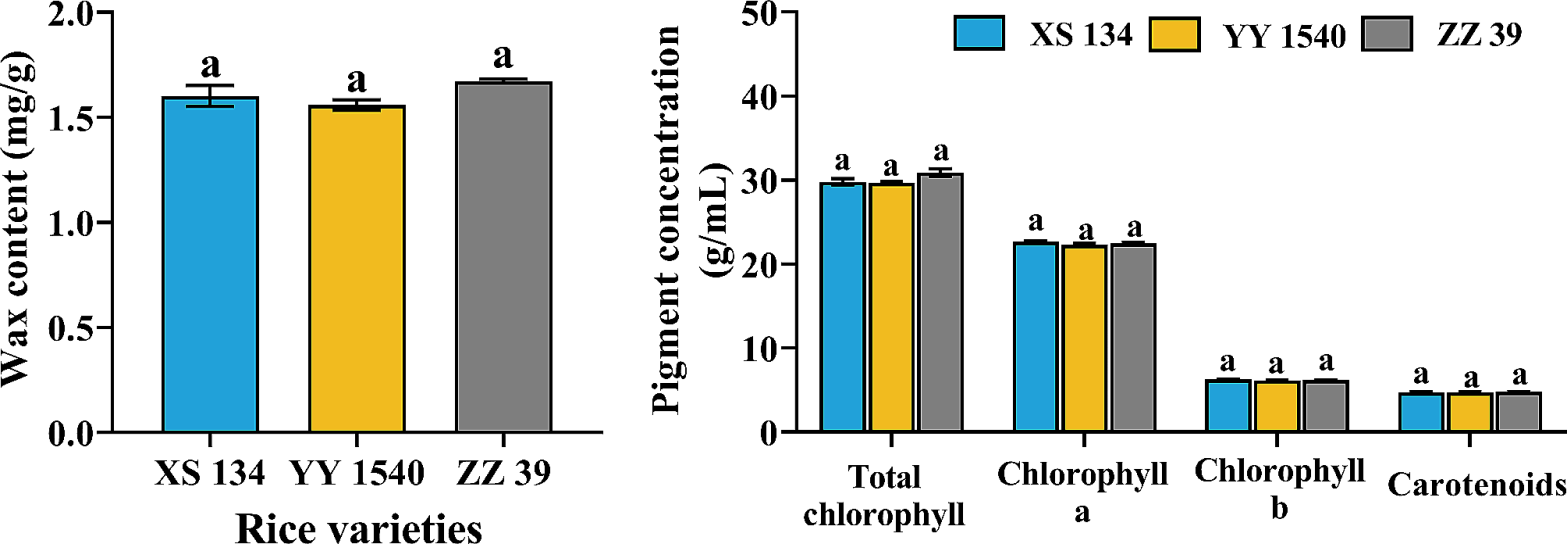
Wax and pigment content in leaves of different rice varieties. Same lowercase letters indicate no significance in different variety rice leaves on wax and pigment content (Tukey’s HSD- *p* > 0.05)

**Fig. 4:**
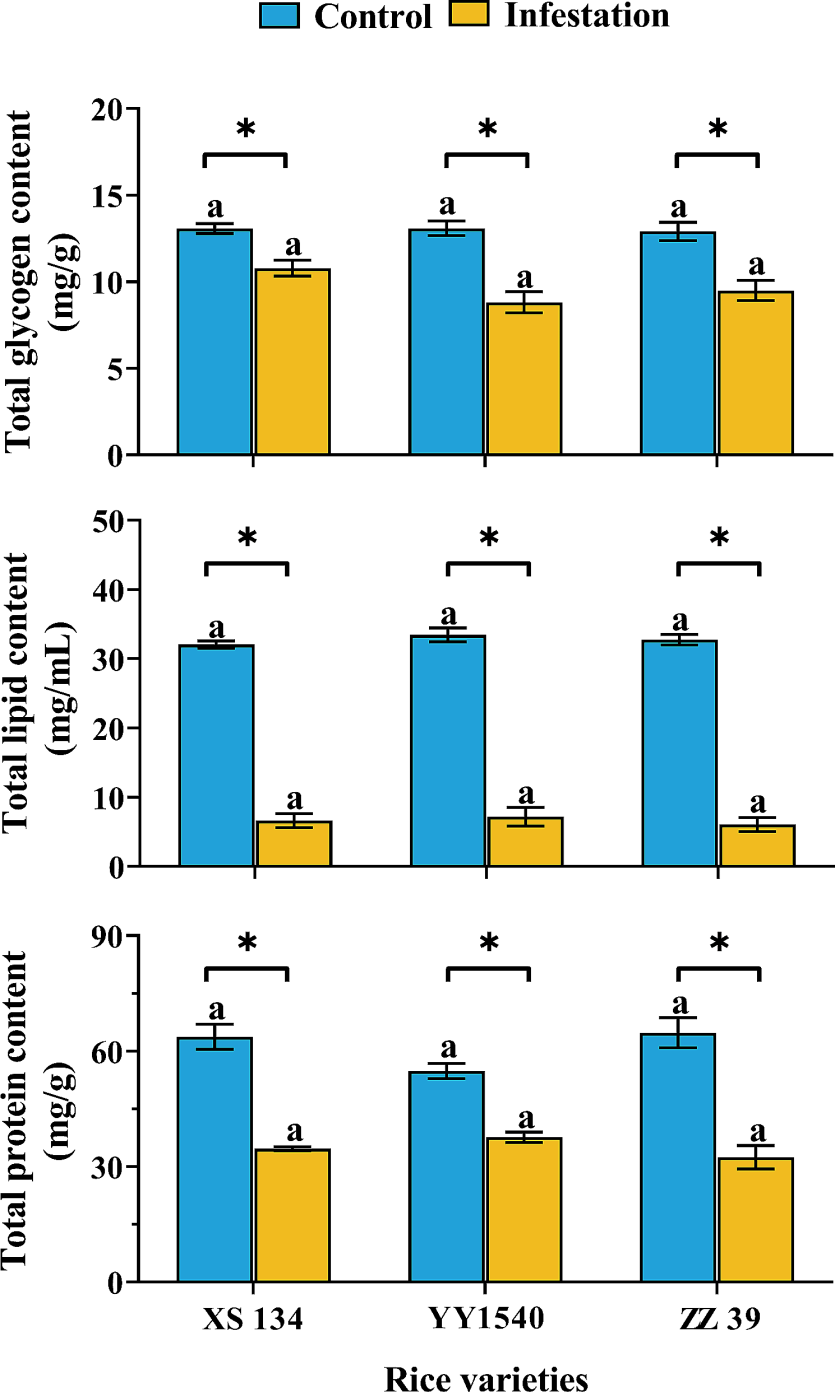
Effects of *C. medinalis* larvae infestation on nutrient content in leaves of different rice varieties. Asterisks indicate significant difference in nutrient content between the leaves of different rice varieties infested and uninfested by *C. medinalis* larvae (*t* test- *p* < 0.05). Same lowercase letters indicate no significance in different variety rice leaves on nutrient content (Tukey’s HSD- *p* > 0.05)

No significant differences were found in the wax content (*F*_*2,6*_ =0.178, *p* = 0.841), pigment concentration (Total chlorophyll: *F*_*2,12*_ = 3.695, *p* = 0.056; chlorophyll a: *F*_*2,12*_ = 1.473, *p* = 0.268; chlorophyll b: *F*_*2,12*_ = 1.085, *p* = 0.369; carotenoids: *F*_*2,12*_ = 0.046, *p* = 0.955), and nutrient content (glycogen: *F*_*2,12*_ = 0.056, *p* = 0.945; total lipid: *F*_*2,12*_ = 0.776, *p* = 0.482; protein: *F*_*2,12*_ = 3.008, *p* = 0.087) of the leaves of the three rice varieties (Figs. 3 and 4). Interestingly, after being infested by *C. medinalis* larvae, total glycogen content (XS134: *F*_*1,8*_ = 17.956, *p* = 0.003; YY1540: *F*_*1,8*_ = 33.736, *p* = 0.001; ZZ39: *F*_*1,8*_ = 18.547, *p* = 0.003), total lipid content (XS134: *F*_*1,8*_ = 492.983, *p* < 0.001; YY1540: *F*_*1,8*_ = 242.313, *p* < 0.001; ZZ39: *F*_*1,8*_ = 452.426, *p* < 0.001) and total protein content (XS134: *F*_*1,8*_ = 77.492, *p* < 0.001; YY1540: *F*_*1,8*_ = 51.855, *p* < 0.001; ZZ39: *F*_*1,8*_ = 42.578, *p* < 0.001) in the leaves of the three rice varieties significantly decreased, albeit no significant difference among different varieties (glycogen: *F*_*2,12*_ = 3.274, *p* = 0.073; lipid: *F*_*2,12*_ = 0.242, *p* = 0.789; protein: *F*_*2,12*_ = 1.774, *p* = 0.211) (Fig. 4).

### Effects of Food Utilization Capability in the 4th instar C. medinalis Feeding on Different Rice Varieties

The ECD (*F*_*2,12*_ = 42.219, *p* < 0.001) and ECI (*F*_*2,12*_ = 49.624, *p* < 0.001) of the 4th instar *C. medinalis* feeding on ZZ39 were significantly lower than those of the other two rice varieties, but their RCR was significantly higher than that of the other two rice varieties (*F*_*2,12*_ = 60.718, *p* < 0.001) (Table 1). However, the AD (*F*_*2,12*_ = 7.282, *p* = 0.008) of larvae fed on YY1540 were significantly lower than those fed on the other two rice varieties, while their RGR was significantly higher (*F*_*2,12*_ = 28.796, *p* < 0.001) (Table 1). The above results indicated that the food utilization capability of the larvae would vary with the rice variety.

**Table 1:** Effects of feeding on different rice varieties on food utilization capability of *C. medinalis* larvae

Food utilization capacity	XS134	YY1540	ZZ39
ECD (%)	25.26 ± 0.41 a	25.87 ± 0.20 a	22.31 ± 0.22 b
ECI (%)	22.91 ± 0.34 a	22.98 ± 0.15 a	20.14 ± 0.14 b
AD (%)	90.71 ± 0.22 a	88.83 ± 0.51 b	90.28 ± 0.30 a
RGR (g/g/d)	0.35 ± 0.01 b	0.40 ± 0.01 a	0.33 ± 0.01 b
RCR (g/g/d)	4.37 ± 0.06 b	4.35 ± 0.03 b	4.97 ± 0.03 a

Different lowercase letters indicate significant differences in food utilization capability among larvae of different rice varieties (Tukey’s HSD- *p* > 0.05).

### Effects of Nutrient Accumulation in the 4th Instar C. medinalis Feeding on Different Rice Varieties

**Fig. 5:**
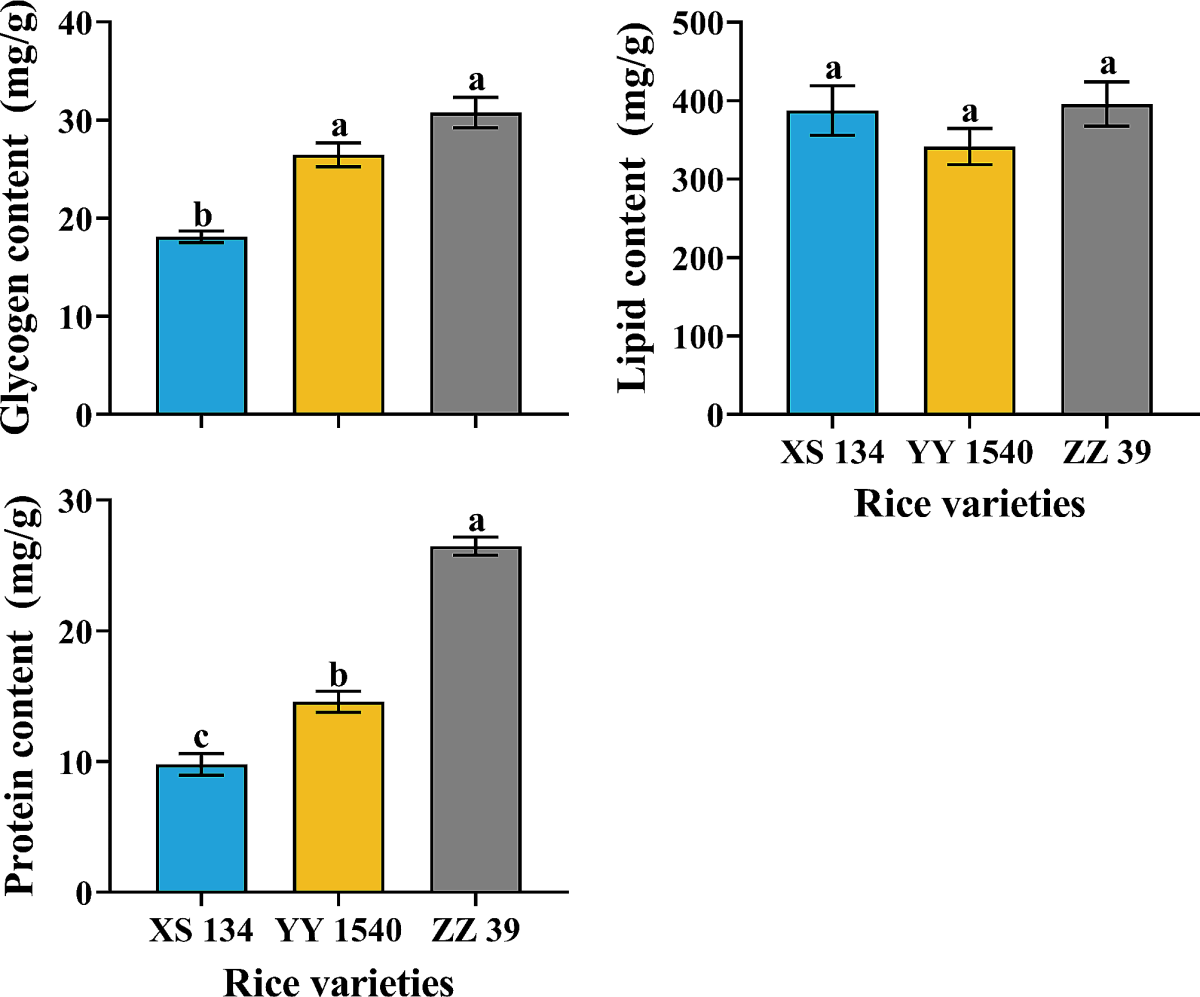
Effects of feeding on different rice varieties on nutrient accumulation of 4th instar *C. medinalis*. Different lowercase letters indicate significant difference in larvae on nutrient accumulation (Tukey’s HSD- *p* > 0.05)

There was no significant difference in the total lipid content of the 4th instar *C. medinalis* feeding on three rice varieties (*F*_*2,12*_ = 1.101, *p* = 0.364). However, significant differences were observed in the accumulation of total glycogen and total protein (Fig. 5). The larvae that fed on XS134 had the lowest total glycogen (*F*_*2,12*_ = 29.295, *p* < 0.001) and protein (*F*_*2,12*_ = 122.387, *p* < 0.001) content in their bodies, while the larvae that fed on ZZ39 had the highest protein accumulation (Fig. 5).

### Effects of 4th instar C. medinalis Infestation on Biochemical Indicators of Leaves of Different Rice Varieties

#### O_2_^−^ Production Rate

**Fig. 6:**
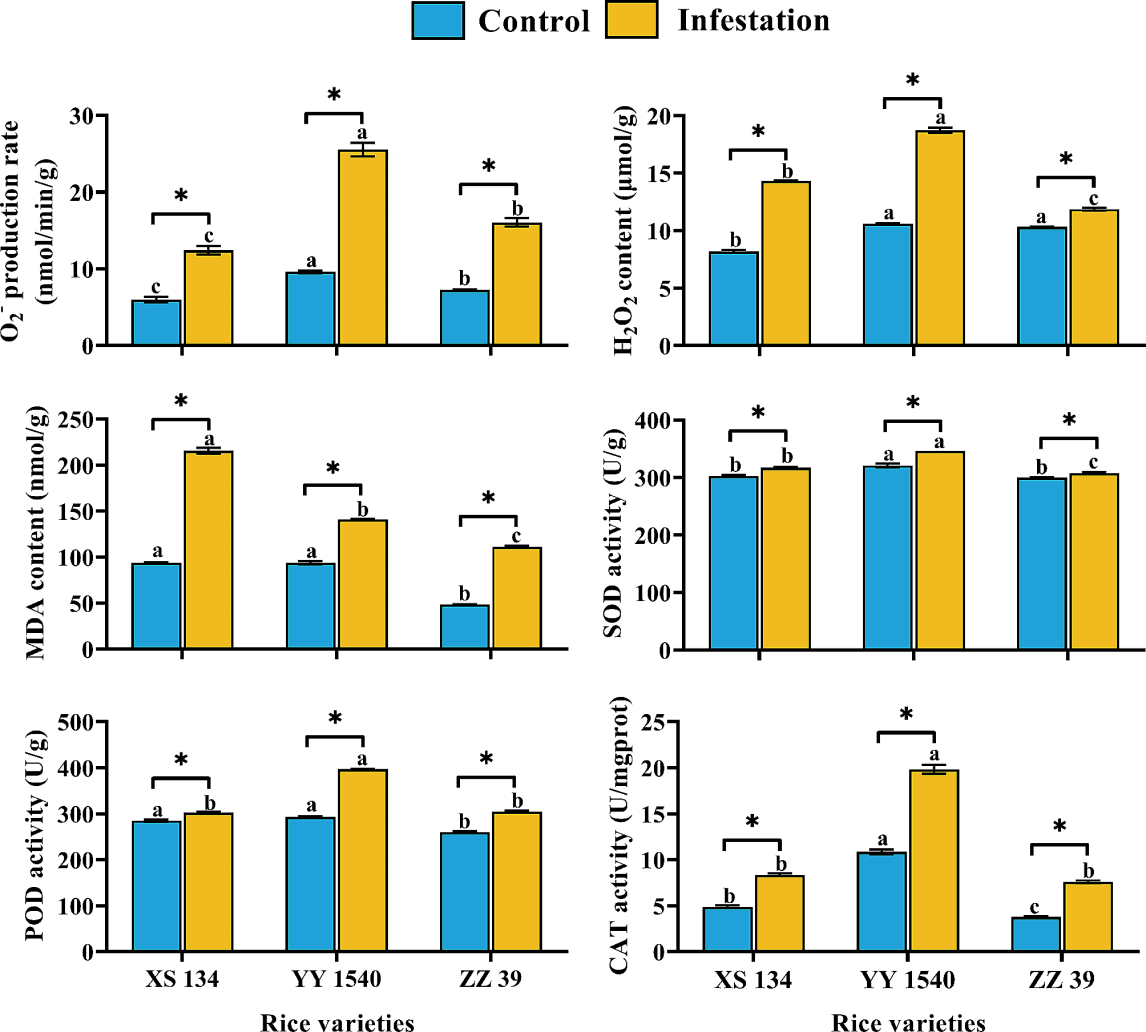
Effects of *C. medinalis* larvae infestation on antioxidant enzyme activity, MDA content, H_2_O_2,_ and O_2_^-^ production rate in leaves of different rice varieties. Different lowercase letters indicate significant differences in leaves on antioxidant enzyme activity, content of MDA, H_2_O_2,_ and O_2_^-^ production rate among leaves of different rice varieties under the same treatment (Tukey’s HSD- *p* < 0.05). Asterisks indicate significant differences in the above indicators between the leaves of different rice varieties infested and uninfested by *C. medinalis* larvae (*t* test- *p* < 0.05)

After being infested by the 4th instar larvae of *C. medinalis*, the O_2_^−^ production rate in the leaves of three rice varieties significantly increased (XS134: *t* = 9.331, df = 4, *p* = 0.001; YY1540: *t* = 18.255, df = 4, *p* < 0.001; ZZ39: *t* = 15.638, df = 4, *p* < 0.001) (Fig. 6). However, regardless of whether the leaves were infested by larvae (*F*_*2,6*_ = 100.061, *p* < 0.001) or not (*F*_*2,6*_ = 58.377, *p* < 0.001), the O_2_^−^ production rate in the leaves of YY1540 was significantly higher than that of the other two rice varieties (Fig. 6).

#### H_2_O_2_ Content

Among the rice leaves that were not infested by the 4th instar *C. medinalis*, the H_2_O_2_ content of XS134 was significantly lower, while there was no significant difference between YY1540 and ZZ39 (*F*_*2,6*_ = 308.502, *p* < 0.001) (Fig. 6). After the infestation of *C. medinalis* larvae, the H_2_O_2_ content in the leaves of three rice varieties was significantly increased (XS134: *t* = 47.513, df = 4, *p* < 0.001; YY1540: *t* = 34.618, df = 4, *p* < 0.001; ZZ39: *t* = 11.287, df = 4, *p* < 0.001). Notably, the H_2_O_2_ content in the leaves of YY1540 after larval infestation was significantly higher but was lower in ZZ39as compared to the other two rice varieties (*F*_*2,6*_ = 497.423, *p* < 0.001) (Fig. 6).

#### MDA Content

No significant differences were observed in the MDA content in YY1540 and XS134 leaves that were not fed by *C. medinalis* larvae (*F*_*2,6*_ = 549.731, *p* < 0.001). The MDA content was significantly increased in the leaves following *C. medinalis* larvae feeding (XS134: *t* = 38.231, df = 4, *p* < 0.001; YY1540: *t* = 23.574, df = 4, *p* < 0.001; ZZ39: *t* = 58.731, df = 4, *p* < 0.001), with XS134 having the highest MDA content, followed by YY1540 and ZZ39 (*F*_*2,6*_ = 756.003, *p* < 0.001) (Fig. 6).

#### Antioxidant Enzyme Activity

In the rice leaves that were not infested by the 4th instar *C. medinalis*, the SOD (*F*_*2,6*_ = 31.442, *p* = 0.001) and CAT (*F*_*2,6*_ = 490.789, *p* < 0.001) activities were significantly higher in YY1540 than the other two varieties, while the POD (*F*_*2,6*_ = 69.325, *p* < 0.001) and CAT activities were dramatically lower in ZZ39 leaves (Fig. 6). The infestation of *C. medinalis* larvae significantly increased the activities of SOD (XS134: *t* = 6.269, df = 4, *p* = 0.003; YY1540: *t* = 8.138, df = 4, *p* = 0.001; ZZ39: *t* = 4.552, df = 4, *p* = 0.011), POD (XS134: *t* = 5.848, df = 4, *p* = 0.004; YY1540: *t* = 58.152, df = 4, *p* < 0.001; ZZ39: *t* = 12.709, df = 4, *p* < 0.001), and CAT (XS134: *t* = 16.795, df = 4, *p* < 0.001; YY1540: *t* = 16.673, df = 4, *p* < 0.001; ZZ39: *t* = 23.484, df = 4, *p* < 0.001) in the leaves of different rice varieties. The highest activity of all three antioxidant enzymes (SOD: *F*_*2,6*_ = 258.725, *p* < 0.001; POD: *F*_*2,6*_ = 758.867, *p* < 0.001; CAT: *F*_*2,6*_ = 520.296, *p* < 0.001) were found in the leaves of YY1540 (Fig. 6).

### Correlation Analysis of Survival Rate, Food Utilization Capability and Nutrient Content of 4th instar C. medinalis, Larval Developmental Duration, and Physiological and Biochemical Indicators in Leaves of Different Rice Varieties after Infestation

**Fig. 7:**
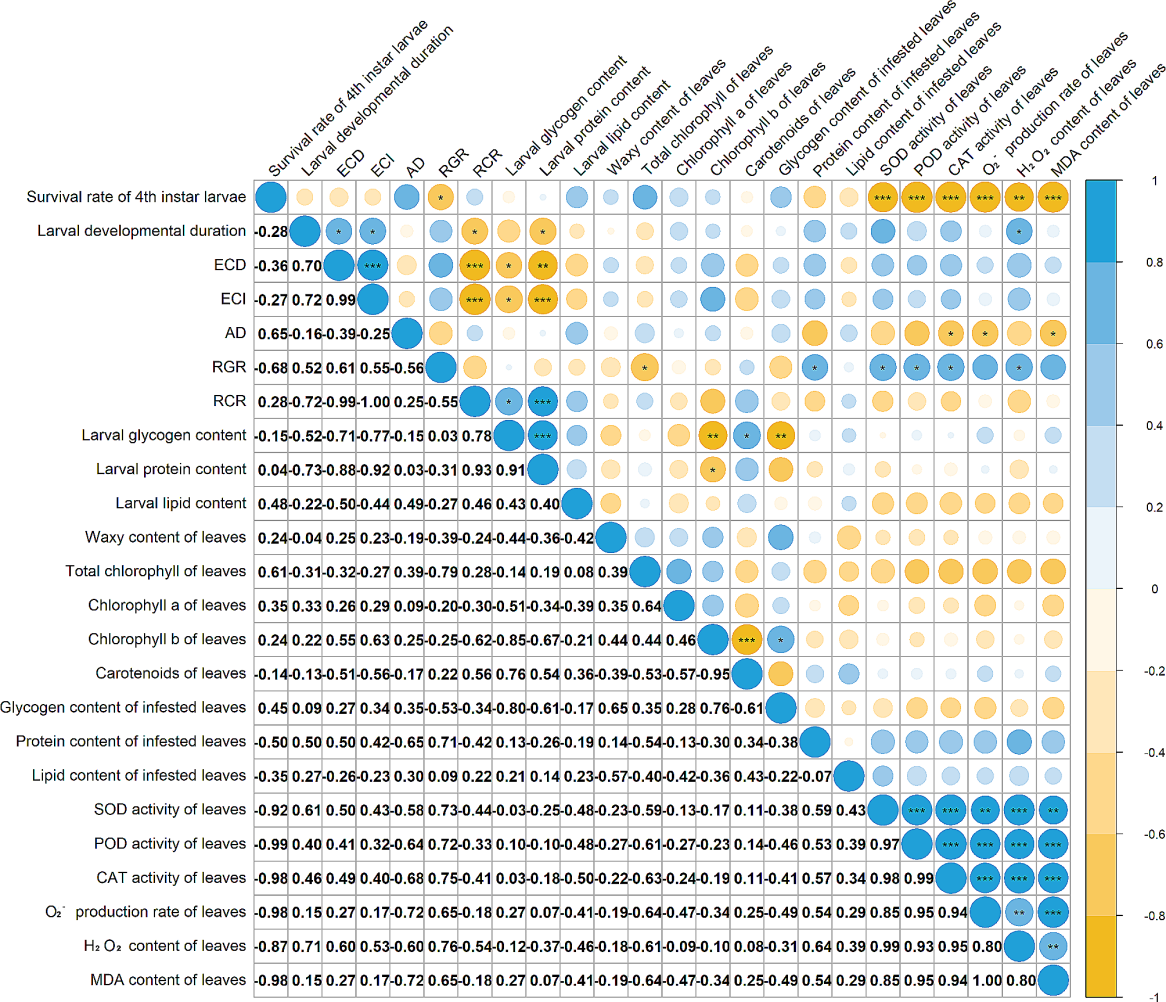
Correlation analysis of survival rate, food utilization capability and nutrient content of 4th instar *C. medinalis*, larval developmental duration, and physiological characteristics and biochemical indicators in leaves of different rice varieties after infestation. The numbers in the bottom left corner represent *r* value. *, ** and *** respectively indicate significant differences at the levels of *p* < 0.05, *p* < 0.01, and *p* < 0.001. The darker the blue color, the stronger the positive correlation, while the darker the yellow color, the stronger the negative correlation. A larger circle indicates a smaller *p* value

The survival and development of the 4th instar *C. medinalis* was closely related to their food utilization capability and rice defense ability. The survival rate of 4th instar larvae was negatively correlated with RGR and all biochemical indicators of rice leaves (O_2_^−^ production rate, H_2_O_2_ content, MDA content, and activities of SOD, POD, CAT) (Fig. 7). The larval developmental period was not only related to their food utilization capability but also to their nutrient accumulation. The development duration of *C. medinalis* larvae was positively correlated with ECD and ECI while negatively correlated with RCR and larval glycogen content (Fig. 7). In addition, the larval development duration was significantly correlated with H_2_O_2_ content of leaves (Fig. 7). Notably, the food utilization capability of larvae to different rice varieties was also related to the rice defense mechanisms. There was a negative correlation between AD and CAT activity, O_2_^−^ production rate, and MDA content of leaves. The RGR was positively correlated with SOD, POD, CAT activity, and H_2_O_2_ content of leaves (Fig. 7). However, the accumulation of nutrients by larvae was only related to their food utilization capability for different rice varieties but not to rice defense ability. The glycogen and protein content of larvae were significantly correlated with ECD, ECI, and RCR but not with the biochemical indicators of rice leaves (Fig. 7).

## Discussion

The resistance of rice crops against insect pests is closely linked with the rice variety (Ab Ghaffar et al. 2011; Antunes et al. 2016). In the current study, the results showed variations in the overall impact of YY1540, ZZ39, and XS134 varieties of rice on the developmental duration, survival rate, food utilization capability, and nutrient accumulation of the *C. medinalis* larvae, indicating that these varieties have different level of resistance against this key pest. The resistance of different rice varieties against insect pests is influenced by their physiological characteristics (Sandhu et al. 2020). However, in this study, no significant differences were found in the wax, pigment, and nutrient content in the leaves of the three rice varieties. This indicates that these parameters were not linked with the differences in the survival and development of larvae after feeding on different rice varieties. Resistant rice varieties may not differ from susceptible varieties in some physiological characteristics, but their defense responses against insect pests are more prominent (Li et al. 2019). Our results show that SOD, POD, CAT activity, H_2_O_2_ content, and O_2_^−^ production rate of different rice varieties infested by *C. medinalis* were closely related to the larvae’s survival, development, and food utilization ability. Among the three rice varieties, the biochemical indicators of YY1540 were highly increased following *C. medinalis* larval infestation. The defense response of different rice varieties affects the growth performance and food utilization efficiency of *C. medinalis* larvae.

The host plant provides nutrients for the growth and development of insects. The survival rate and developmental duration of larvae are important parameters for measuring the suitability of insects on the host plant (Azidah and Sofian-Azirun 2006; Chen et al. 2018; Sedighi et al. 2017; Wu et al. 2021). Zhang et al. (2011) and Soufbaf et al. (2010) reported that the survival rate of the larvae of *Spodoptera exigua* and *Plutella xylostella* were significantly decreased while the larval developmental periods were increased on host plants with low fitness. The total developmental period of *C. medinalis* larvae was prolonged after feeding on XS134 and YY1540. Increasing the larval duration to obtain the necessary nutrients for growth and development may be a response to poor growth conditions (Li et al. 2021). Moreover, 1st to 3rd instar *C. medinalis* survival rate significantly decreased after feeding on YY1540. These results indicated that YY1540 was highly unfavorable for the occurrence of *C. medinalis* populations. In previous studies, it was found that leaf wax, pigment, and nutrient content of some rice varieties were related to their resistance to *C. medinalis* (Ge et al. 2013; Xu et al. 2010; Wang et al. 2008). However, leaf physiological characteristics vary depending on rice varieties (Xu et al. 2010). We found no differences in leaf wax, pigment, and nutrition content among the three rice varieties we studied. These physiological indicators were not significantly correlated with the survival and development of larvae. This indicates that the physiological characteristics of these three rice varieties did not directly influence the growth and developmental changes of larvae after feeding. When insects attack plants, their photosynthetic and primary metabolic genes are inhibited, possibly allocating more resources to producing defense compounds (Hermsmeier et al. 2001; Rayapuram and Baldwin 2006). This shift between growth and defense responses can alter the plant’s nutritional status (Hui et al. 2013) and may influence insect growth and development. For example, the content of nutrients in plants damaged by *P. Xylostella* and *Dendroctonus armandi* significantly decreased (Pu and Chen 2007; Yin et al. 2012). Our study also yielded the same results, showing a significant decrease in leaf nutrient content among the three rice varieties after feeding on *C. medinalis* larvae. Nevertheless, there was no significant difference among varieties in the leaf nutrient content following larval feeding. These results indicated that the alternation of nutrient content due to larval feeding does not sufficiently explain the variability of larval growth and development period observed among varieties. In addition, there was no significant alteration in feeding preferences among the 1st to 4th instar of *C. medinalis* towards three different rice varieties, which was not associated with the differences in survival rate and developmental duration of larvae. Insects have no particular preference for host plants throughout their development and growth stages (Fei et al. 2017). Juvenile lepidoptera larvae require more nutrition for their development. However, they directly consume food due to low mobility without seeking alternative host plants (Barton 2007; Quintero and Bowers 2018). Notably, the 5th instar *C. medinalis* had a lower preference for XX134. In *Chironomus calliraphus* Goeldi, it was found that larvae entering the pupal stage prefer animal food and algae that enable them to grow rapidly (Banegas and Rocha 2023). There was no significant difference in nutritional content among the three rice varieties, but the larvae had significantly lower levels of glycogen and protein content after feeding on XS134. These findings suggest that XS134 might produce defense substances that hinder the digestion and absorption of glycogen and protein after infestation. As a result, the larvae’s transition into the pupal stage is delayed, prolonging their overall development period. However, this has only a minor effect on their survival rate. This may be the reason why the 5th instar larvae had less preference for XS134.

Although we did not find any variations in nutrient content among different rice varieties, there were differences in nutrient accumulation among the 4th instar *C. medinalis* that fed on these three rice varieties. The accumulation of nutrients in insects is related to their digestion and utilization ability (Pendreño et al. 2006). Similar to the larvae’s survival, growth, and development results, the food utilization capability of *C. medinalis* larvae feeding on YY1540 was significantly different from the other two rice varieties. The ECI and ECD of 4th instar larvae fed on YY1540 were higher, but their AD and RCR were lower. This phenomenon of feeding on different host plants resulting in differences in food utilization capacity is similar in many Lepidoptera insects (Wang et al. 2015; Zhang et al. 2009). Although the 4th instar larvae feeding on YY1540 had strong food utilization capability, their protein accumulation in the body was low. Insects exhibit physiological and behavioral adaptations in response to plant defense compounds, resulting in alterations such as modifying the food consumption rate or enhancing their digestive efficiency. These adaptations ultimately lead to increased energy allocation from food intake to body mass (Kholghahmadi et al. 2023; Rayapuram and Baldwin 2006). After infestation by *C. medinalis*, YY1540 may produce defense substances such as protease inhibitors that inhibit larval feeding and decrease the digestibility of leaf proteins (Cheah et al. 2020; Bolter and Jongsma 1997), resulting in lower protein accumulation. However, to maintain normal development, larvae may increase their growth rate by increasing their intake of other nutrients and digesting food conversion efficiency. Protein is a vital component for the composition of body tissues, regulation of physiological functions, and provision of energy. The energy supply is strongly linked to the development, metabolism, and survival of insects (Geiser and Winzerling 2011; Saikhedkar et al. 2015; Tepass 2012). The lower survival rates and slower development observed in larvae that consume YY1540 may be attributed to reduced protein accumulation. This can be attributed to the high demand for protein in insects and the costly allocation of amino acids and energy towards synthesizing growth and development-related enzymes (Hinks et al. 1991). Therefore, we hypothesized that the variations in defensive compounds among the three rice varieties following infestation by *C. medinalis* could account for differences in larval survival, growth, development, food utilization, and nutrient accumulation.

ROS, as an early signaling molecule, can activate the subsequent defense response of plants, mainly including O_2_^−^ and H_2_O_2_ (Mittler 2017; Sewelam et al. 2016). To a certain extent, the production rate of O_2_^−^ and the content of H_2_O_2_ reflect the plant’s defense response to biological stimuli (Dey and Bhattacharjee 2020; Li and Yi 2012; Ribeiro et al. 2017). MDA can amplify the effect of ROS (Sakihama et al. 2002; Zhu et al. 2021). As expected, the rice variety YY1540 had the most significant inhibitory impact on the survival and growth of C. medinalis larvae. This was accompanied by the highest O_2_^−^ production rate and H_2_O_2_ content following infestation, suggesting that YY1540 possesses a more vigorous defense response against *C. medinalis* larvae infestation. Interestingly, in the leaves of XS134 fed by *C. medinalis*, the O_2_^−^ production rate was very low, with H_2_O_2_ content second only to YY1540, but MDA content was the highest. Plants often undergo membrane lipid peroxidation when subjected to stress, and the final decomposition product of this process is MDA, which can reflect the degree of stress damage to plants (Katerova et al. 2021; Laxa et al. 2019). XS134 had a relatively weak defense response when invaded by larvae. Nevertheless, it experienced the most significant level of damage. SOD has a key role in defense against ROS, converting ROS into H_2_O_2_. POD and CAT decompose H_2_O_2_ into H_2_O, thereby eliminating the damage caused by ROS (Apel and Hirt 2004; Ge et al. 2019). SOD, POD, and CAT activities in the leaves of YY1540 significantly increased among the three rice varieties following larvae infestation. This indicated that YY1540 has a protective effect on the damage caused by the infestation of *C. medinalis* larvae. In addition, correlation analysis shows that the survival and development of *C. medinalis* larvae were closely related to their food utilization capability and production and elimination of ROS. ROS plays a crucial role in various aspects of plant defense (Marslin et al. 2017). ROS accumulation can induce plants to exhibit stimulated antioxidant enzyme activity activities such as phenylalanine ammonia lyase (PAL) and glutathione reductase, leading to an increase in the production of secondary metabolites such as flavonoids and tannins (Gautam et al. 2023; Ho et al. 2020; Han and Yuan 2004). These secondary metabolites have negative impacts on insect populations, such as inhibiting insect development, increasing mortality, reducing feeding efficiency, and decreasing protein utilization efficiency (Gautam et al. 2023; Mierziak et al. 2014; Zheng et al. 2022). Cheah et al. (2020) found that the accumulation of PAL potentially enhances the resistance of a *C. medinalis*-resistant rice variety (Qingliu) by priming the biosynthesis of essential flavonoids. In our study, the differences in survival rate, growth and development, food utilization capacity, and nutrient accumulation among the three rice varieties may be related to the flavonoids induced by ROS. Furthermore, ROS can also serve as signals for other messengers such as jasmonic acid (JA) and ethylene (ET) to indirectly regulate secondary metabolism, and lead to the induction of plant defense downstream in the form of defense proteins to herbivores (Baxter et al. 2014; Gautam et al. 2023; Malook et al. 2022; Zhang et al. 2016). These defense proteins were shown to enhance anti-nutritive or anti-digestive effects in insects, such as protein inhibitors, α-amylase inhibitors, lectins and polyphenol oxidases (Fürstenberg-Hägg et al. 2013). Anti-digestive proteins limit the enzyme conversion rate of ingested food, whereas anti-nutritive proteins limit the utilization of food by altering physical availability and/or chemical identity, ultimately affecting insect development and survival (Duffey and Stout 1996; Mithöfer and Boland 2012). In the rice variety japonica XiuShui 11, it was found that the attack of *C. medinalis* larvae led to JA biosynthe-sis activation in leaves, promoting the accumulation of trypsin proteinase inhibitor and phenolamides, thereby reducing larval performance (Zhuang et al. 2022). Three rice varieties produce different levels of ROS in response to the feeding of *C. medinalis*, which may trigger specific signal transduction pathways and induce the production of different defense proteins, thereby affecting the food utilization capacity and nutrient accumulation of larvae. The multiple roles of ROS in direct and indirect insect resistance defense may have resulted in three rice varieties exhibiting varying degrees of resistance to *C. medinalis*. After infestation by *C. medinalis*, more ROS induced more or specific defense substances in YY1540 may affect the digestion and absorption of nutrients by the larvae. This might lead to changes in the energy accumulation of the larvae, ultimately resulting in the lowest survival rate and prolonged development period of the larvae feeding on YY1540. However, we did not investigate what specific defensive substances produced by YY1540 that distinguish it from the other two rice varieties in terms of their ability to inhibit the survival and growth of *C. medinalis* larvae. Additionally, the exact involvement of ROS in developing distinct defense mechanisms among rice varieties remains unclear.

## Conclusion

In summary, YY1540 had a more significant inhibitory effect on the growth and development of *C. medinalis* larvae among the three rice varieties. This was not due to differences in the wax, chlorophyll, and nutrient content of the leaves among the three rice varieties, nor to the feeding preference of the larvae. YY1540 had the highest O_2_^−^ production rate, H_2_O_2_ content, and SOD, POD, and CAT activities in the leaves after *C. medinalis* larvae infestation. These defense reactions were significantly correlated with the survival, development, and food utilization capability of larvae, which may lead to differences in the ability of larvae to utilize food and accumulate nutrients, further affecting their survival, growth and development. However, this study only analyzed the differences in some physiological characteristics and enzyme activities related to ROS production and elimination among rice varieties. It clarified that YY1540 had a more robust defense response to the infestation of *C. medinalis* and its correlation with ROS production and elimination. Based on our findings, we have raised new questions about the defense mechanisms of different rice varieties against the infestation of *C. medinalis* larvae: What factors contribute to the variations in the rate and quantity of ROS production among different rice varieties? Has this difference caused YY1540 to produce defense substances that are different from the other two rice varieties? How do these defense substances affect the survival, growth, and development of *C. medinalis* larvae? It is crucial to clarify these issues to regulate ROS signals and subsequent defense responses, which could ultimately help control *C. medinalis*. Additionally, this will facilitate the development of rice cultivars resistant to this key pest.

### Electronic Supplementary Material

Below is the link to the electronic supplementary material.

**Additional File 1: Supplementary Figure S1.** The leaf rolling rate of *C. medinalis* on three rice varieties

## Data Availability

The data that support the findings of this study are available within the paper, and more information, if required, can be requested to the corresponding author.

## References

[CR2] Abbasi AR, Hajirezaei M, Hofius D, Sonnewald U, Voll LM (2007). Specific roles of alpha- and gamma-tocopherol in abiotic stress responses of transgenic tobacco. Plant Physiol.

[CR1] Ab Ghaffar MB, Pritchard J, Ford-Lloyd B (2011). Brown planthopper *(N. lugens* Stal) feeding behaviour on rice germplasm as an indicator of resistance. PLoS ONE.

[CR3] Alamgir KM, Hojo Y, Christeller JT, Fukumoto K, Isshiki R, Shinya T, Baldwin IT, Galis I (2016). Systematic analysis of rice (*Oryza sativa*) metabolic responses to herbivory. Plant Cell Environ.

[CR4] Alinia F, Ghareyazie B, Rubia L, Bennett J, Cohen MB (2000). Effect of plant age, larval age, and fertilizer treatment on resistance of a cry1Ab-transformed aromatic rice to lepidopterous stem borers and foliage feeders. J Econ Entomol.

[CR5] Amb MK, Ahluwalia AS (2016). Allelopathy: potential role to achieve new milestones in rice cultivation. Rice Sci.

[CR6] Antunes C, Mendes R, Lima A, Barros G, Fields P, Da Costa LB, Rodrigues JC, Silva MJ, Correia AM, Carvalho MO (2016). Resistance of rice varieties to the stored-product insect, *Sitophilus zeamais* (Coleoptera: Curculionidae). J Econ Entomol.

[CR7] Apel K, Hirt H (2004). Reactive oxygen species: metabolism, oxidative stress, and signal transduction. Annu Rev Plant Biol.

[CR8] Azidah AA, Sofian-Azirun M (2006). Life history of *Spodoptera exigua* (Lepidoptera: Noctuidae) on various host plants. Bull Entomol Res.

[CR95] Banegas BP, Rocha L (2023) *Chironomus calligraphus* Goeldi, 1905 (Diptera: Chironomidae) larvae: feeding habits, growth, and maturation. Neotrop Entomol 52(3):431–441. 10.1007/s13744-023-01029-1 10.1007/s13744-023-01029-1 36826745

[CR9] Barton KE (2007). Early ontogenetic patterns in chemical defense in Plantago (Plantaginaceae): genetic variation and trade-offs. Am J Bot.

[CR10] Baxter A, Mittler R, Suzuki N (2014). ROS as key players in plant stress signalling. J Exp Bot.

[CR11] Bhavanam S, Stout MJ (2022). Varietal resistance and chemical ecology of the rice stink bug, *Oebalus pugnax*, on rice, *Oryza sativa*. Plants.

[CR12] Bittner N, Trauer-Kizilelma U, Hilker M (2017). Early plant defence against insect attack: involvement of reactive oxygen species in plant responses to insect egg deposition. Planta.

[CR13] Bolter C, Jongsma MA (1997). The adaptation of insects to plant protease inhibitors. J Insect Physiol.

[CR14] Cheah BH, Lin HH, Chien HJ, Liao CT, Liu LD, Lai CC, Lin YF, Chuang WP (2020). SWATH-MS-based quantitative proteomics reveals a uniquely intricate defense response in *Cnaphalocrocis medinalis*-resistant rice. Sci Rep.

[CR15] Cheah BH, Nadarajah K, Divate MD, Wickneswari R (2015). Identification of four functionally important microRNA families with contrasting differential expression profiles between drought-tolerant and susceptible rice leaf at vegetative stage. BMC Genomics.

[CR16] Chen GM, Chi H, Wang RC, Wang YP, Xu YY, Li XD, Yin P, Zheng FQ (2018). Demography and uncertainty of population growth of *Conogethes punctiferalis* (Lepidoptera: Crambidae) reared on five host plants with discussion on some life history statistics. J Econ Entomol.

[CR17] Cheng XY, Zhu LL, He GC (2013). Towards understanding of molecular interactions between rice and the brown planthopper. Mol Plant.

[CR18] Cui Q, Wu J, Li DZ, Wu F, Han RL, Huang JH, Hu SQ (2023). Changes of coloration and pigment compositions during leaf development of Osmanthus fragrans colour group cultivar. J Nanjing for Univ.

[CR20] Desneux N, Decourtye A, Delpuech J-M (2007). The sublethal effects of pesticides on beneficial arthropods. Annu Rev Entomol.

[CR19] Dey N, Bhattacharjee S (2020). Accumulation of polyphenolic compounds and osmolytes under dehydration stress and their implication in redox regulation in four indigenous aromatic rice cultivars. Rice Sci.

[CR21] Dong Y, Fang X, Yang Y, Xue GP, Chen X, Zhang W, Wang X, Yu C, Zhou J, Mei Q, Fang W, Yan C, Chen J (2017). Comparative proteomic analysis of susceptible and resistant rice plants during early infestation by small brown planthopper. Front Plant Sci.

[CR22] Duffey SS, Stout MJ (1996). Antinutritive and toxic components of plant defence against insects. Arch Insect Biochem Physiol.

[CR23] Fei M, Harvey JA, Yin Y, Gols R (2017). Oviposition preference for young plants by the large cabbage butterfly (*Pieris brassicae*) does not strongly correlate with caterpillar performance. J Chem Ecol.

[CR24] Fürstenberg-Hägg J, Zagrobelny M, Bak S (2013). Plant defense against insect herbivores. Int J Mol Sci.

[CR25] Gautam H, Sharma A, Trivedi PK (2023). The role of flavonols in insect resistance and stress response. Curr Opin Plant Biol.

[CR28] Geiser DL, Winzerling JJ (2011). Insect transferrins: multifunctional proteins. Biochim Biophys Acta.

[CR26] Ge LQ, Wang F, Wu JC (2013). Resistance of different rice varieties to *Cnaphalocrocis Medinalis* Güenée (Lepidoptera: Pyralidae) larvae infestation. J Yangzhou Univ.

[CR27] Ge Y, Tang Q, Li C, Duan B, Li X, Wei M, Li J (2019). Acibenzolar-S-methyl treatment enhances antioxidant ability and phenylpropanoid pathway of blueberries during low temperature storage. LWT.

[CR29] Gill SS, Tuteja N (2010). Reactive oxygen species and antioxidant machinery in abiotic stress tolerance in crop plants. Plant Physiol Biochem.

[CR30] Guo JW, Cui Y, Lin PJ, Zhai BP, Lu ZX, Chapman JW, Hu G (2022). Male nutritional status does not impact the reproductive potential of female *Cnaphalocrocis medinalis* moths under conditions of nutrient shortage. Insect Sci.

[CR31] Guo JW, Yang F, Li P, Liu XD, Wu QL, Hu G, Zhai BP (2019). Female bias in an immigratory population of *Cnaphalocrocis medinalis* moths based on field surveys and laboratory tests. Sci Rep.

[CR32] Guo WP, Liao CT, Chuang WP (2019). Defensive responses of rice cultivars resistant to *Cnaphalocrocis Medinalis* (Lepidoptera: Crambidae). Anthropod-Plant Inte.

[CR33] Han GJ, Liu Q, Li CM, Xu B, Xu J (2021). Transcriptome sequencing reveals *Cnaphalocrocis medinalis* against baculovirus infection by oxidative stress. Mol Immunol.

[CR34] Han RB, Yuan YJ (2004). Oxidative burst in suspension culture of *Taxus cuspidata* induced by a laminar shear stress in short-term. Biotechnol Prog.

[CR35] Hermsmeier D, Schittko U, Baldwin IT (2001). Molecular interactions between the specialist herbivore *Manduca sexta* (Lepidoptera, Sphingidae) and its natural host *Nicotiana attenuata*. I. large-scale changes in the accumulation of growth- and defense-related plant mRNAs. Plant Physiol.

[CR36] Hinks CF, Cheeseman M, Erlandson MA, Olfert O, Westcott ND (1991). The effects of kochia, wheat and oats on digestive proteinases and the protein economy of adult grasshoppers, *Melanoplus sanguinipes*. J Insect Physiol.

[CR37] Ho TT, Murthy HN, Park SY (2020). Methyl jasmonate induced oxidative stress and accumulation of secondary metabolites in plant cell and organ cultures. Int J Mol Sci.

[CR39] Hui YY, Wen ZQ, Xia LX (2013). The relationship between the contents of nutrients and tannins in different cotton varieties and their resistance to *Apolygus Lucorum*. Sci Agric Sin.

[CR38] Hu LF, Ye M, Kuai P, Ye MF, Erb M, Lou YG (2018). OsLRR-RLK1, an early responsive leucine-rich repeat receptor-like kinase, initiates rice defense responses against a chewing herbivore. New Phytol.

[CR40] Hussain HA, Hussain S, Khaliq A, Ashraf U, Anjum SA, Men S, Wang L (2018). Chilling and drought stresses in crop plants: implications, cross talk, and potential management opportunities. Front Plant Sci.

[CR41] Jin P, Chen J, Zhan H, Huang S, Wang J, Shu Y (2020). Accumulation and excretion of zinc and their effects on growth and food utilization of *Spodoptera litura* (Lepidoptera: Noctuidae). Ecotoxicol Environ Saf.

[CR42] Katerova Z, Sergiev I, Todorova D, Shopova E, Dimitrova L, Brankova L (2021). Physiological responses of wheat seedlings to soil waterlogging applied after treatment with selective herbicide. Plants.

[CR43] Kerchev PI, Fenton B, Foyer CH, Hancock RD (2012). Plant responses to insect herbivory: interactions between photosynthesis, reactive oxygen species and hormonal signalling pathways. Plant Cell Environ.

[CR44] Kholghahmadi M, Karimi-Malati A, Jalali Sendi J (2023) Ecophysiological responses of individually and group reared *Cydalima Perspectalis* (Lepidoptera: Crambidae) to alkaloid-containing host plants. Environ Entomol 52(3):426–435. 10.1093/ee/nvad017 10.1093/ee/nvad017 36988446

[CR45] Kumar K, Mandal SN, Neelam K, de Los Reyes BG (2022). MicroRNA-mediated host defense mechanisms against pathogens and herbivores in rice: balancing gains from genetic resistance with trade-offs to productivity potential. BMC Plant Biol.

[CR46] Laxa M, Liebthal M, Telman W, Chibani K, Dietz KJ (2019). The role of the plant antioxidant system in drought tolerance. Antioxidants.

[CR47] Li L, Yi HL (2012). Effect of sulfur dioxide on ROS production, gene expression and antioxidant enzyme activity in Arabidopsis plants. Plant Physiol Biochem.

[CR48] Li Q, Kuo YW, Lin KH, Huang W, Deng C, Yeh KW, Chen SP (2021). Piriformospora indica colonization increases the growth, development, and herbivory resistance of sweet potato (*Ipomoea batatas* L). Plant Cell Rep.

[CR50] Liu F, Cheng JJ, Jiang T, Su W, Xu S (2012). Selectiveness of *Cnaphalocrocis medinalis* to host plants. Rice Sci.

[CR51] Liu Q, Hallerman E, Peng Y, Li Y (2016). Development of Bt rice and bt maize in China and their efficacy in target pest control. Int J Mol Sci.

[CR52] Liu X, Williams CE, Nemacheck JA, Wang H, Subramanyam S, Zheng C, Chen MS (2010). Reactive oxygen species are involved in plant defense against a gall midge. Plant Physiol.

[CR49] Li Y, Huang YF, Huang SH, Kuang YH, Tung CW, Liao CT, Chuang WP (2019). Genomic and phenotypic evaluation of rice susceptible check TN1 collected in Taiwan. Bot Stud.

[CR53] Malook SU, Maqbool S, Hafeez M, Karunarathna SC, Suwannarach N (2022). Molecular and biochemical mechanisms of elicitors in pest resistance. Life.

[CR54] Marslin G, Sheeba CJ, Franklin G (2017). Nanoparticles alter secondary metabolism in plants via ROS burst. Front Plant Sci.

[CR55] Mierziak J, Kostyn K, Kulma A (2014). Flavonoids as important molecules of plant interactions with the environment. Molecules.

[CR57] Miller G, Shulaev V, Mittler R (2008). Reactive oxygen signaling and abiotic stress. Physiol Plant.

[CR56] Mithöfer A, Boland W (2012). Plant defense against herbivores: chemical aspects. Annu Rev Plant Biol.

[CR58] Mittler R (2017). ROS are good. Trends Plant Sci.

[CR59] Mittler R, Zandalinas SI, Fichman Y, Van Breusegem F (2022). Reactive oxygen species signalling in plant stress responses. Nat Rev Mol Cell Biol.

[CR60] Nanda S, Yuan SY, Lai FX, Wang WX, Fu Q, Wan PJ (2020). Identification and analysis of miRNAs in IR56 rice in response to BPH infestations of different virulence levels. Sci Rep.

[CR61] Nouman W, Anwar F, Gull T, Newton A, Rosa E, Domínguez-Perles R (2016). Profiling of polyphenolics, nutrients and antioxidant potential of germplasm’s leaves from seven cultivars of *Moringa oleifera* Lam. Ind Crop Prod.

[CR62] Nouman W, Basra SMA, Yasmeen A, Gull T, Hussain SB, Zubair M, Gul R (2014). Seed priming improves the emergence potential, growth and antioxidant system of *Moringa oleifera* under saline conditions. Plant Growth Regul.

[CR63] Ojha A, Zhang WQ (2019). A comparative study of microbial community and dynamics of Asaia in the brown planthopper from susceptible and resistant rice varieties. BMC Microbiol.

[CR64] Peñalver-Cruz A, Horgan FG (2022). Interactions between rice resistance to planthoppers and honeydew-related egg parasitism under varying levels of nitrogenous fertilizer. Insects.

[CR65] Pendreño Y, González-Párraga P, Conesa S, Martínez-Esparza M, Aguinaga A, Hernández JA, Argüelles JC (2006). The cellular resistance against oxidative stress (H_2_O_2_) is independent of neutral trehalase (Ntc1p) activity in *Candida albicans*. FEMS Yeast Res.

[CR66] Pu XJ, Chen H (2007). Relations between attacking of *Dendroctonus armandi* and nutrition and resistance material of host trees (*Pinus armandi*). J Northwest F Univ.

[CR67] Quintero C, Bowers MD (2018). Plant and herbivore ontogeny interact to shape the preference, performance and chemical defense of a specialist herbivore. Oecologia.

[CR68] Rayapuram C, Baldwin IT (2006). Using nutritional indices to study LOX3-dependent insect resistance. Plant Cell Environ.

[CR69] Ribeiro CW, Korbes AP, Garighan JA, Jardim-Messeder D, Carvalho FEL, Sousa RHV, Caverzan A, Teixeira FK, Silveira JAG, Margis-Pinheiro M (2017). Rice peroxisomal ascorbate peroxidase knockdown affects ROS signaling and triggers early leaf senescence. Plant Sci.

[CR71] Saikhedkar N, Summanwar A, Joshi R, Giri A (2015). Cathepsins of lepidopteran insects: aspects and prospects. Insect Biochem Mol Biol.

[CR70] Sakihama Y, Cohen MF, Grace SC, Yamasaki H (2002). Plant phenolic antioxidant and prooxidant activities: phenolics-induced oxidative damage mediated by metals in plants. Toxicology.

[CR72] Sandhu RK, Sarao PS, Kumari N (2020). Biochemical responses associated with resistance to *Nilaparvata lugens* (Stål) in wild rice accessions. Rice Sci.

[CR73] Sedighi L, Ranjbar Aghdam H, Imani S, Shojai M (2017). Age-stage two-sex life table analysis of *Sesamia nonagrioides* (Lep.: Noctuidae) reared on different host plants. Arch Phytopathol Plant Protect.

[CR74] Sewelam N, Kazan K, Schenk PM (2016). Global plant stress signaling: reactive oxygen species at the cross-road. Front Plant Sci.

[CR75] Shangguan XX, Zhang J, Liu BF, Zhao Y, Wang HY, Wang ZZ, Guo JP, Rao WW, Jing SL, Guan W, Ma YH, Wu Y, Hu L, Chen RZ, Du B, Zhu LL, Yu DZ, He GC (2018). A mucin-like protein of planthopper is required for feeding and induces immunity response in plants. Plant Physiol.

[CR76] Soufbaf M, Fathipour Y, Karimzadeh J, Zalucki MP (2010). Bottom-up effect of different host plants on *Plutella xylostella* (Lepidoptera: Plutellidae): a life-table study on canola. J Econ Entomol.

[CR77] Sun Y, Liu ST, Ling Y, Wang L, Ni H, Guo D, Dong BB, Huang Q, Long LP, Zhang S, Wu SF, Gao CF (2023). Insecticide resistance monitoring of *Cnaphalocrocis Medinalis* (Lepidoptera: Pyralidae) and its mechanism to chlorantraniliprole. Pest Manag Sci.

[CR78] Tang FH, Lenzen M, McBratney A, Maggi F (2021). Risk of pesticide pollution at the global scale. Nat Geosci.

[CR79] Tepass U (2012). The apical polarity protein network in *Drosophila* epithelial cells: regulation of polarity, junctions, morphogenesis, cell growth, and survival. Annu Rev Cell Dev Biol.

[CR80] Thorpe GW, Reodica M, Davies MJ, Heeren G, Jarolim S, Pillay B, Breitenbach M, Higgins VJ, Dawes IW (2013). Superoxide radicals have a protective role during H_2_O_2_ stress. Mol Biol Cell.

[CR81] Wang QQ, Wang L, Li KB, Cao YZ, Yin J, Xiao C (2015). Influences of different host plants on the nutrition and digestive enzymes of *Loxostege Sticticali*. Plant Protect.

[CR82] Wang QX, Xu L, Wu JC (2008). Physical and biochemical mechanisms of resistance of different rice varieties to the rice leaffolder *Cnaphalocrocis Medinalis* (Lepidoptera: Pyralidae). Acta Entomol Sin.

[CR83] Wu LH, Zhou C, Long GY, Yang XB, Wei ZY, Liao YJ, Yang H, Hu CX (2021). Fitness of fall armyworm, *Spodoptera frugiperda* to three solanaceous vegetables. J Integr Agric.

[CR84] Xu J, Wang QX, Wu JC (2010). Resistance of cultivated rice varieties to *Cnaphalocrocis Medinalis* (Lepidoptera: Pyralidae). J Econ Entomol.

[CR85] Yadava CP, Santaram G, Israel P, Kalode MB (1972). Life-history of rice leaf-roller, *Cnaphalocrocis Medinalis* Guenée (Lepidoptera: Pyralidae) and its reaction to some rice varieties and grasses. Indian J Agric Sci.

[CR86] Yin F, Feng X, Zhang DY, Li ZY, Lin QS, Hu ZD, Chen HY (2012). Contents changes of protein and sugar in host plants after damaged by the diamondback moth, *Plutella xylostella* (L.) (Lepidoptera: Plutellidae). J Environ Entomol.

[CR87] Zhang B, Liu H, Helen HS, Wang JJ (2011). Effect of host plants on development, fecundity and enzyme activity of *Spodoptera exigua* (Hübner) (Lepidoptera: Noctuidae). Agric Sci Chin.

[CR88] Zhang HB, Li A, Zhang ZJ, Huang ZJ, Lu PL, Zhang DY, Liu XM, Zhang ZF, Huang RF (2016). Ethylene response factor TERF1, regulated by ETHYLENE-INSENSITIVE3-like factors, functions in reactive oxygen species (ROS) scavenging in tobacco (*Nicotiana tabacum* L). Sci Rep.

[CR89] Zhang JF, Xue QZ (2004). The activity dynamics of main protective enzymes in rice plants under feeding stresses of *Sogatella furcifera* and *Nilaparvata lugens*. Sci Agric Sin.

[CR90] Zhang N, Guo JY, Wan FH, Wu G (2009). Effects of three kinds of host plants on development and some digestive enzyme activities of beet armyworm, *Spodoptera exigua*. J Plant Prot.

[CR91] Zhao XY, Guo JW, Lu YH, Sun TY, Tian JC, Huang JL, Xu HX, Wang ZL, Lu ZX (2022). Reference genes for expression analysis using RT-qPCR in *Cnaphalocrocis Medinalis* (Lepidoptera: Pyralidae). Insects.

[CR92] Zheng SC, Liu WJ, Luo JY, Wang LS, Zhu XZ, Gao XK, Hua HX, Cui JJ (2022). *Helicoverpa Armigera* herbivory negatively impacts *Aphis gossypii* populations via inducible metabolic changes. Pest Manag Sci.

[CR94] Zhuang YQ, Wang XJ, Llorca LC, Lu J, Lou YG, Li R (2022). Role of jasmonate signaling in rice resistance to the leaf folder *Cnaphalocrocis Medinalis*. Plant Mol Biol.

[CR93] Zhu CQ, Hu WJ, Cao XC, Zhu LF, Kong YL, Jin QY, Shen GX, Wang WP, Zhang H, Zhang JH (2021). Physiological and proteomic analyses reveal effects of putrescine-alleviated aluminum toxicity in rice roots. Rice Sci.

