# Supplementary material for: Defense Responses of Different Rice Varieties Affect Growth Performance and Food Utilization of Cnaphalocrocis medinalis Larvae
Source: Rice (N Y). 2024 Jan 20;17:9. doi: 10.1186/s12284-024-00683-2 (PMC10799839; doi:10.1186/s12284-024-00683-2)
Supplement: Supplementary file 1 — Additional File 1: Supplementary Figure S1. The leaf rolling rate of C. medinalis on three rice varieties [file 12284_2024_683_MOESM1_ESM.docx]

Defense responses of different rice varieties affect growth performance and food utilization of *Cnaphalocrocis medinalis* larvae

Xiaoyu Zhao ^1,2^, Hongxing Xu ^1^, Yajun Yang ^1^, Tianyi Sun ^1,3^, Farman Ullah ^1^, Pingyang Zhu ^4^, Yanhui Lu ^1^, Jianlei Huang ^5^, Zhengliang Wang ^2^, Zhongxian Lu ^1*^ and Jiawen Guo ^1*^

1. State Key Laboratory for Managing Biotic and Chemical Threats to the Quality and Safety of Agro-Products, Institute of Plant Protection and Microbiology, Zhejiang Academy of Agricultural Sciences, Hangzhou 310021, China;

2. College of Life Sciences, China Jiliang University, Hangzhou, Zhejiang 310018, China;

3. College of Plant Protection, Nanjing Agricultural University, Nanjing 210095, China;

4. College of Life Sciences, Zhejiang Normal University, Jinhua 321004, China;

5. College of Agriculture and Forestry, Hebei North University, Zhangjiakou 075000, China

^*^ Corresponding authors:

GUO Jiawen: Address: No. 198 Shiqiao Road, Hangzhou, Zhejiang Province, China, Tel.: +86-(0)-0571-88045127, E-mail: [guojiawen91@126.com](mailto:guojiawen91@126.com);

Lu Zhongxian: Address: No. 198 Shiqiao Road, Hangzhou, Zhejiang Province, China, Tel.: +86-(0)571-86404077, E-mail: [luzxmh@163.com](mailto:luzxmh@163.com)

**Supplementary information**


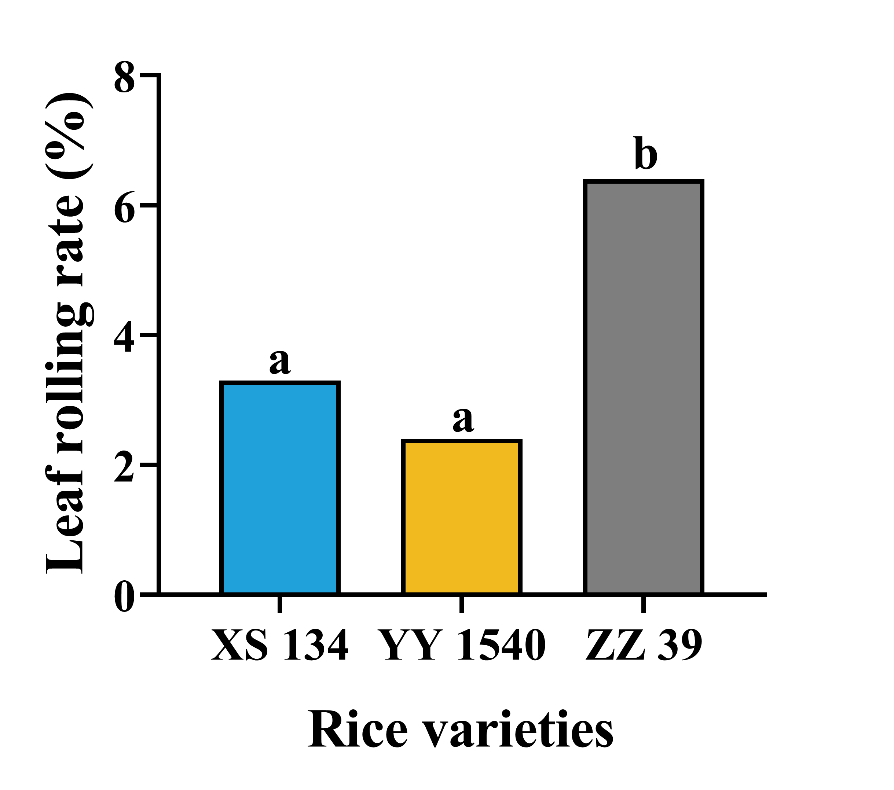


**Fig. S1.** The leaf rolling rate of *C. medinalis* on three rice varieties. The three rice varieties are Zhongzao39 (ZZ39), Xiushui 134 (XS134), and Yongyou 1540 (YY1540). The planting time and development status of the three rice varieties are consistent. The Chi-squared test analysis of data shows that the occurrence of *C. medinalis* on three rice varieties is significantly different, with the lowest leaf roller rate of YY1540 and the highest leaf roller rate of ZZ39.
